# Adaptive laboratory evolution to obtain furfural tolerant *Saccharomyces cerevisiae* for bioethanol production and the underlying mechanism

**DOI:** 10.3389/fmicb.2023.1333777

**Published:** 2024-01-04

**Authors:** Lan Yao, Youpiao Jia, Qingyan Zhang, Xueyun Zheng, Haitao Yang, Jun Dai, Xiong Chen

**Affiliations:** ^1^Key Laboratory of Fermentation Engineering (Ministry of Education), Cooperative Innovation Center of Industrial Fermentation (Ministry of Education and Hubei Province), College of Bioengineering, Hubei University of Technology, Wuhan, China; ^2^Hubei Provincial Key Laboratory of Green Materials for Light Industry, Hubei University of Technology, Wuhan, China

**Keywords:** adaptive laboratory evolution, *Saccharomyces cerevisiae*, furfural tolerance, bioethanol, CRISPR/Cas9

## Abstract

**Introduction:**

Furfural, a main inhibitor produced during pretreatment of lignocellulose, has shown inhibitory effects on *S. cerevisiae*.

**Method:**

In the present study, new strains named 12–1 with enhanced resistance to furfural were obtained through adaptive laboratory evolution, which exhibited a shortened lag phase by 36 h, and an increased ethanol conversion rate by 6.67% under 4 g/L furfural.

**Results and Discussion:**

To further explore the mechanism of enhanced furfural tolerance, ADR1_1802 mutant was constructed by CRISPR/Cas9 technology, based on whole genome re-sequencing data. The results indicated that the time when ADR1_1802 begin to grow was shortened by 20 h compared with reference strain (*S. cerevisiae* CEN.PK113-5D) when furfural was 4 g/L. Additionally, the transcription levels of *GRE2* and *ADH6* in ADR1_ 1802 mutant were increased by 53.69 and 44.95%, respectively, according to real-time fluorescence quantitative PCR analysis. These findings suggest that the enhanced furfural tolerance of mutant is due to accelerated furfural degradation. Importance: Renewable carbon worldwide is vital to achieve “zero carbon” target. Bioethanol obtained from biomass is one of them. To make bioethanol price competitive to fossil fuel, higher ethanol yield is necessary, therefore, monosaccharide produced during biomass pretreatment should be effectively converted to ethanol by *Saccharomyces cerevisiae*. However, inhibitors formed by glucose or xylose oxidation could make ethanol yield lower. Thus, inhibitor tolerant *Saccharomyces cerevisiae* is important to this process. As one of the main component of pretreatment hydrolysate, furfural shows obvious impact on growth and ethanol production of *Saccharomyces cerevisiae*. To get furfural tolerant *Saccharomyces cerevisiae* and find the underlying mechanism, adaptive laboratory evolution and CRISPR/Cas9 technology were applied in the present study

## 1 Introduction

The wide development and application of lignocellulosic materials in biofuel industry represent a pivotal approach to provide sufficient alternative fuels for fossile fuels (Lin and Lu, 2021). Nevertheless, pretreatment before fermentation is a necessity to enhance hexose and pentose yield from lignocellulose during enzymatic hydrolysis process, as it generates a substantial amount of by-products simultaneously that severely hinder the growth and metabolism of microorganisms (Rabemanolontsoa and Saka, 2016). The content and type of generated inhibitors are closely related with applied pretreatment method. During dilute acid pretreatment, most of hemicellulose is dissolved to generate hexose and pentose, which are degraded further to form inhibitors for microorganism, such as 5-HMF, furfural, formic acid and levulinic acid. The concentrations of these inhibitors are enhanced with increased concentration of applied acid. After alkali pretreatment, phenolic inhibitors in the hydrolysate are significantly increased, of which ferulic acid shows strong cytotoxic effect on cells (Alves et al., 2020). The content of inhibitors varies for different lignocellulose hydrolysates. The acetic acid concentration in rice straw and softwood hydrolysate ranges from 0.49 to 6.5 g/L. The content of furfural, 5-HMF and vanillin after hydrothermal pretreatment is 0.2–2.0, 0.1–1.0, and 0.5–2.0 g/L, respectively (He et al., 2016).

Liu et al. investigated the impact of various inhibitors derived from biomass on the growth of *Rhodosporon*, including furfural, 5-hydroxymethylfurfural, acetic acid, vanillic acid, and vanillin. The results demonstrated that furfural showed the strongest inhibitory effect, followed by vanillin and 5-HMF (Liu et al., 2021). Moreover, it has been proven that aldehydes can be detoxified by *Saccharomyces cerevisiae in situ* to corresponding alcohols. This reduction reaction is catalyzed by NAD (P)H-dependent aldehyde reductase (Wang et al., 2018). *Saccharomyces cerevisiae* can convert 100% HMF to furfural at a concentration of 60 mM, and 100% furfural to furfuryl alcohol at 30 mM (Liu et al., 2005).

Therefore, enhancing the tolerance of microorganisms to inhibitors is a crucial approach. Recently, a strain improvement strategy called “adaptive laboratory evolution” (ALE) has gained widespread application (Wu et al., 2022). Adaptive laboratory evolution is the process of obtaining new microbial phenotypes through natural selection. Compared with other technologies, ALE is not restricted by physiological mechanisms behind microbial phenotypes to achieve improvement. The combination of ALE and DNA sequencing technology has been widely applied to identify key gene mutations (Sandberg et al., 2019). But it requires a longer time for forced selection of specific phenotypes, and negative mutations may also occur (Hemansi et al., 2022). Through adaptive laboratory evolution, a robust mutant with enhanced inhibitor tolerance for *Kluyveromyces marxianus* 1727 was obtained in mixed inhibitors with increased concentrations by Du and his coworkers. The mutant showed significantly increased uptake rates of acetate, formate, furfural, and vanillin (Du et al., 2022). Earlier studies revealed that the ethanol yield of *Zymomonas mobilis* was severely inhibited by the phenolic aldehyde produced by lignocellulosic pretreatment. But following a process of laboratory adaptive evolution, the obtained *Zymomonas mobilis* Z198 showed a significant increase in the conversion rate of vanillin by 6.3 times, and an increase in ethanol production by 21.6% (Yan et al., 2021).

Researchers tried to obtain enhanced stress tolerant strains for ethanol fermentation, but the underlying mechanism are still not fully understood. Several genes related to furfural tolerance of yeast were identified already, including *SFP1*, *sigI*, *araR*, *YAP1*, *GSH1*, *GLR1*, *YNL134C*, *YML131W*, *ADH6* (Petersson et al., 2006; Kim and Hahn, 2013; Li et al., 2014; Zhao et al., 2015; Chen et al., 2016; Alves et al., 2020). ALE can improve the robustness of microorganisms to specific selection pressures, and can be useful to find genes responsible for further metabolic engineering (Jin et al., 2019).

Characteristics of *Saccharomyces cerevisiae* includes low fermentation cost, high ethanol production, high safety, and is widely applied in industrial fermentation (Yu and Zhang, 2004). Microorganism with higher tolerance, higher ethanol production, and clear genetic background is a better choice for exploration of genes related to furfural tolerance enhancement and underlying mechanisms. A deeper understanding of the furfural tolerance mechanism of yeast can reduce the production cost of ethanol (Sharma et al., 2022). In this study, ALE was first applied to obtain genetically stable and resistant strains to furfural stress, followed by complete genome sequencing and comparative genomic analysis to identify changes in genes related to enhanced furfural stress. Furthermore, a mutant strain was constructed using CRISPR/Cas9 to explore the potential mechanism of increased furfural tolerance. This study has shed light on the molecular mechanisms related to furfural tolerance in *Saccharomyces cerevisiae* through genome sequencing and reverse engineering.

## 2 Materials and methods

### 2.1 Strain and cultivation conditions

*Saccharomyces cerevisiae* CEN.PK113-7D, *S. cerevisiae CEN.PK113-5D*, *S. cerevisiae* 12–1 (obtained after ALE) were maintained in slants. *Escherichia coli* JM110 was applied for plasmid construction.

Precultures were grown overnight at 30°C and 200 rpm in YEPD until the exponential phase (about 12 h).

### 2.2 Methods

#### 2.2.1 Adaptive laboratory evolution

**Figure 1:**
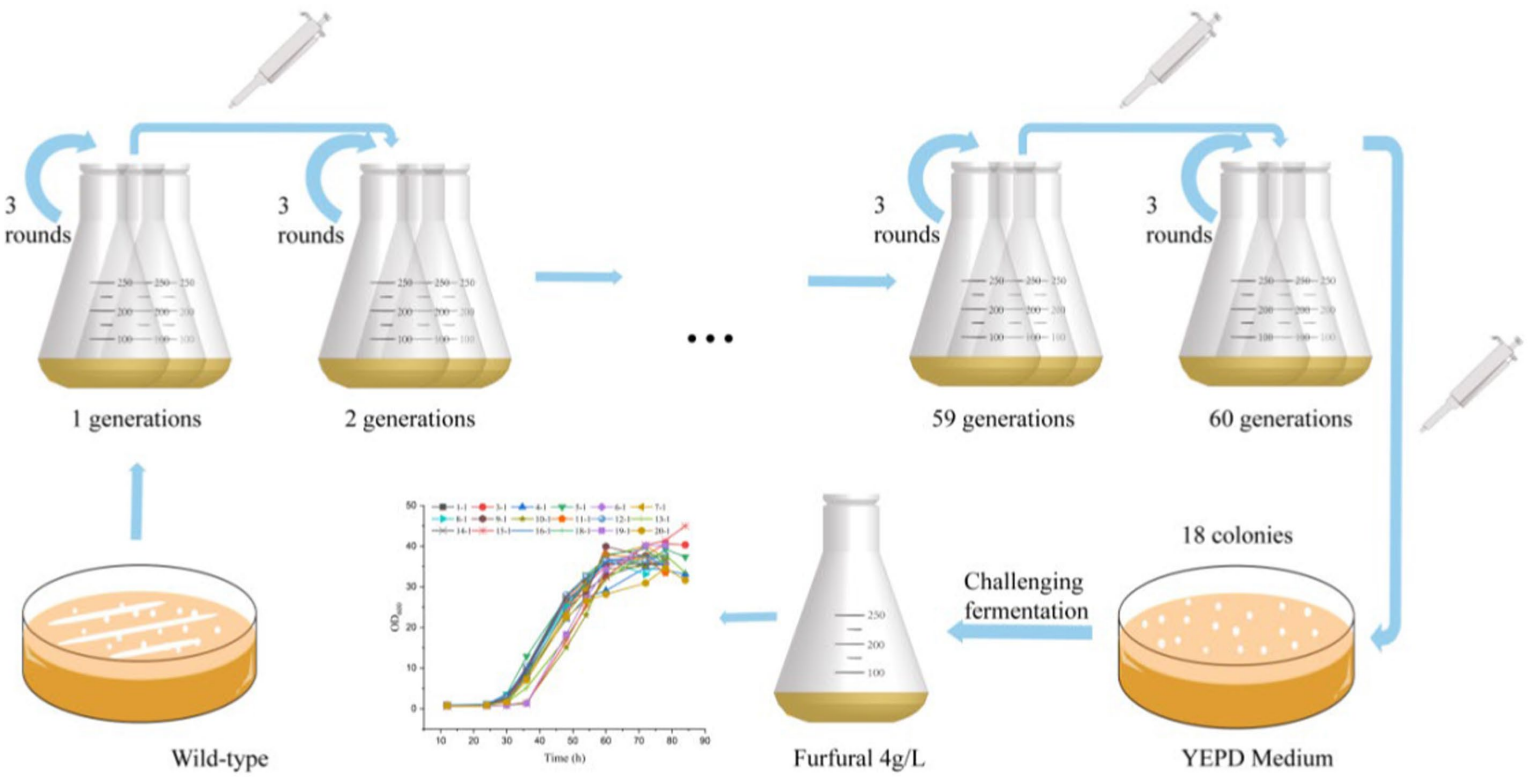
Flow diagram of adaptive laboratory evolution.

The wild type *S. cerevisiae* CEN.PK113-7D strain was subjected to serial batch culture in YEPD with increased furfural stress (from 1 to 3.8 g/L), as shown in Figure 1. 1 g/L furfural was the initial stress concentration. When the yeast grows to 80% of the maximum OD600 value, inoculation is carried out. The culture is transferred to fresh 50 mL medium with OD600 of 0.5. The furfural concentration is increased by 0.2 g/L each time, the passage is repeated three times in each cycle. After more than 20 rounds of passage in 98 days, concentration of furfural reached 3.8 g/L finally. After 98 days of adaptive laboratory evolution, the culture medium was spread on YEPD medium containing 1 g/L furfural for preliminary screening.

#### 2.2.2 Determination of biomass, glucose, ethanol, and furfural

The concentration of biomass, glucose, ethanol and furfural was same as previously published studies (Dai et al., 2020; Jia et al., 2022).

#### 2.2.3 Spot assay of the viability of yeast cells

0, 0.5, 0.8, 1, and 1.5 g/L of furfural were added to the YEPD culture medium that had just been sterilized. The OD600 of wild-type strain and adapted strain was diluted to 0.6, which then was diluted to 10^−1^, 10^−2^, 10^−3^. Take 1 μL of each concentration and place them in the corresponding marked area of the plate and kept in a 30°C incubator for 60 h.

#### 2.2.4 DNA extraction, sequencing, and analysis

The *Saccharomyces cerevisiae CEN.PK113-7D* and *Saccharomyces cerevisiae* 12–1 were cultivated overnight in YEPD medium. The yeast cells were obtained after centrifuge at 12,000 rpm for 1 min. Omega Fungal DNA Kit was applied for DNA extraction (D3390-02, Tiangen Biochemical Technology Co., Ltd.). Quantitative purification was performed using a TBS-380 fluorometer (Turner BioSystems Inc., Sunnyvale, CA). Then, the DNA was sent to Shanghai Majorbio Biopharm Biotechnology Co., Ltd. for sequencing.

The analysis process was referred to published literatures (Xia et al., 2022).

#### 2.2.5 Strain construction by CRISPR/Cas9

CRISPR/Cas9 technology was applied to construct a mutated ADR1 based on pML104 plasmid, the process was shown in Supplementary Figure S1. Primers used in the construction of strains were listed in Table 1.

**Table 1:** Primers used in the construction of strains.

Primer name	Sequence (5′–3′)
ADR1gRNA-f	gatcattaaatagctttgattctagttttagagctag
ADR1gRNA-r	ctagctctaaaactagaatcaaagctatttaat
ADR1-UF	gcatatgtcggcgctaacacgaattctaagaacgcttca
ADR1-UR	aagcactctaatgcacatatttctcattggttcattaaatagctttgattctatactatgcgatggctg
ADR1-DF	cagccatcgcatagtatagaatcaaagctatttaatgaaccaatgagaaatatgtgcattagagtgctt
ADR1-DR	ctccaaataatcagcaactagagcgaacatgaagctcaatattagggattgc
T3	gcaattaaccctcactaaagg

Ten microliter preculture of *E.coli* JM110 containing pML104 plasmid (stored in our laboratory) was incubated at 37°C and 200 rpm for 12–16 h in LB medium containing 100 μg/mL ampicillin sodium. pML104 plasmid was extracted following the FastPure@ Plasma Mini Kit instructions (Nanjing Novozan Biotechnology Co., Ltd.). Then linearized pML104 plasmid was constructed for the cloning of sgRNA.

gRNA was designed near the mutation site, which was then hybridized as described previously (Xia et al., 2022). Then it was directly ligated into linearized pML104 plasmids. The recombinant plasmid pML104-adr1gRNA was obtained and transformed to select positive transformants. Insertion validation of gRNA into the pML104 plasmid was confirmed using T3 primers.

Upstream (825 bp) and downstream (612 bp) homologous arms of *ADR1* were obtained by PCR amplification of ADR1-UF/ADR1-UR and ADRI-DF/ADR1-DR from *Saccharomyces cerevisiae* CEN.PK113-5D genomic DNA. After that, a donor fragment contains a mutation in the ADRI gene at position 1802 (A changes to G) was obtained. After transformation, PCR was used to identify positive colonies were tested by PCR, which was confirmed by sequencing (Laughery et al., 2015).

#### 2.2.6 Determination of glycerol, catalase, and superoxide dismutase

Take 1 mL of fermentation broth, centrifuge at 12,000 r/min for 5 min to get the cells, which then was washed 3 times with deionized water. The ratio of 10^4^ cells to the volume of the extraction solution (mL) was 500–1,000:1. Ultrasound (power 20%, ultrasound for 3 s, interval of 10 s, repeat 30 times) was applied to get the cell lysate, and the supernatant for detection of catalase activity and superoxide dismutase was obtained after centrifuge at 4°C for 10 min at 8,000 r/min.

Take 1 mL of fermentation broth, centrifuge at 12,000 r/min for 10 min to get the cells, which then was washed 3 times with deionized water. After adding 1 mL of ultrapure water and an appropriate amount of glass beads, the disrupted cells were obtained after shaking and crushing with a vortex meter for 20 min. The supernatant obtained after centrifuge at 12,000 r/min for 10 min was applied for intracellular glycerol content determination.

The concentration of glycerol is detected by a glycerol detection kit (E1002, Beijing Pulilai Gene Technology Co., Ltd.). Catalase activity was measured by a catalase detection kit (A007-1-1, Nanjing Jiancheng Biotechnology Research Institute). Superoxide dismutase was measured by a superoxide dismutase detection kit (A001-3-1, Nanjing Jiancheng Biotechnology Research Institute).

#### 2.2.7 Fluorescence quantitative PCR

*S. cerevisiae* CEN.PK113-5D and ADR1_ 1802 were incubated at 30°C at 200 rpm. When the OD_600_ reached approximately 1.0, 4 g/L of furfural was added to the medium, and the time was defined as 0 h. After incubation for 2 h, the cells were collected by centrifuging at 4,000 r/min at room temperature for 2 min. Cells obtained from cultures free of furfural at 0 and 2 h were taken as controls. The primers used for qRT-PCR analysis were listed in Table 2. Total RNA was isolated according to previously described protocol (Liu and Slininger, 2007), and then purified using the RNA clean kit (Tiangen Biotechnology Co., Ltd., Beijing, China). The reverse transcription reaction was carried out using the procedure described by Lewis et al. (2009). HiScript II QRT SuperMix (Norwich Biotechnology Co., Ltd.) and ChamQ universal SYBR qPCR Master Mix (Norwich Biotechnology Co., Ltd.) were applied for qRT-PCR. The qRT-PCR data were analyzed using the method described by Liu et al. (2009). The reference gene used for quantitative PCR is *ENO1*.

**Table 2:** Primers for qRT-PCR.

Gene	Primer name	Sequence
ENO1	ENO1-F	tcttcaaggacggtaagtacga
	ENO1R	acagtcaagtcatcagcaacaa
ALD4	ALD4-F	atggcgaacgattctgaa
	ALD4-R	ggcttattgatgaccttactc
GRE2	GRE2-F	acgcctactgtggttcta
	GRE2-R	gttcgcaagatgtgttcaa
GRE3	GRE3-F	gtgttgatgaaggcttgatt
	GRE3-R	ggaccgaaggaggagtaa
ARI1	ARI1-F	gaggagagttggaataaggata
	ARI1-R	cggattgatagtggatagtgt
YPR1	YPR1-F	aagccgttggtgtctcta
	YPR1-R	tgtggtagcaatggatgaat
ADH6	ADH6-F	gataccaccaagaccaacta
	ADH6-R	ggctgctccactattatgt

#### 2.2.8 Statistical analysis

OriginPro 2019b software (Northampton, MA, United States) was used for diagrams drawing. GraphPad Prism 8 and Statistica 23.0 software (SPSS Inc., Chicago, IL, United States) were applied for statistical analysis. All experiments were conducted with triplicate.

## 3 Results

### 3.1 Screening of mutant strains after adaptive laboratory evolution

The Adaptive Laboratory Evolution (ALE) strategy is used to obtain robust *Saccharomyces cerevisiae* cells that can tolerate high concentrations of furfural inhibitors. *S. cerevisiae* CEN PK 113-7D is widely used in metabolic engineering and systems biology research, and it shows excellent growth characteristics in industrial production. It is widely used in metabolic and evolutionary engineering research, such as fermentation of pentose, the production of ethanol, lactic acid and pyruvate (Nijkamp et al., 2012). Thus, it was applied in the present study as the starting strain. Prior to ALE, it is important to determine the appropriate initial furfural concentration that will inhibit yeast cells to a certain extent, but not severely damage yeast cells. Our preliminary results showed that 1 g/L of furfural was determined as the initial concentration for evolution.

The endpoint concentration of furfural reached 3.8 g/L. A total of 18 colonies were isolated, which were applied for further analysis of furfural tolerance. As shown in Supplementary Figure S3, under the stress of 4 g/L furfural, the cells of wild-type strains were elongated larger, flattened, irregular. The surface was unsmooth, and vacuoles could be observed. While cells of mutant strains were regular, round, plump. The surface was smooth, and a small amount of sprouting could be observed.

**Figure 2:**
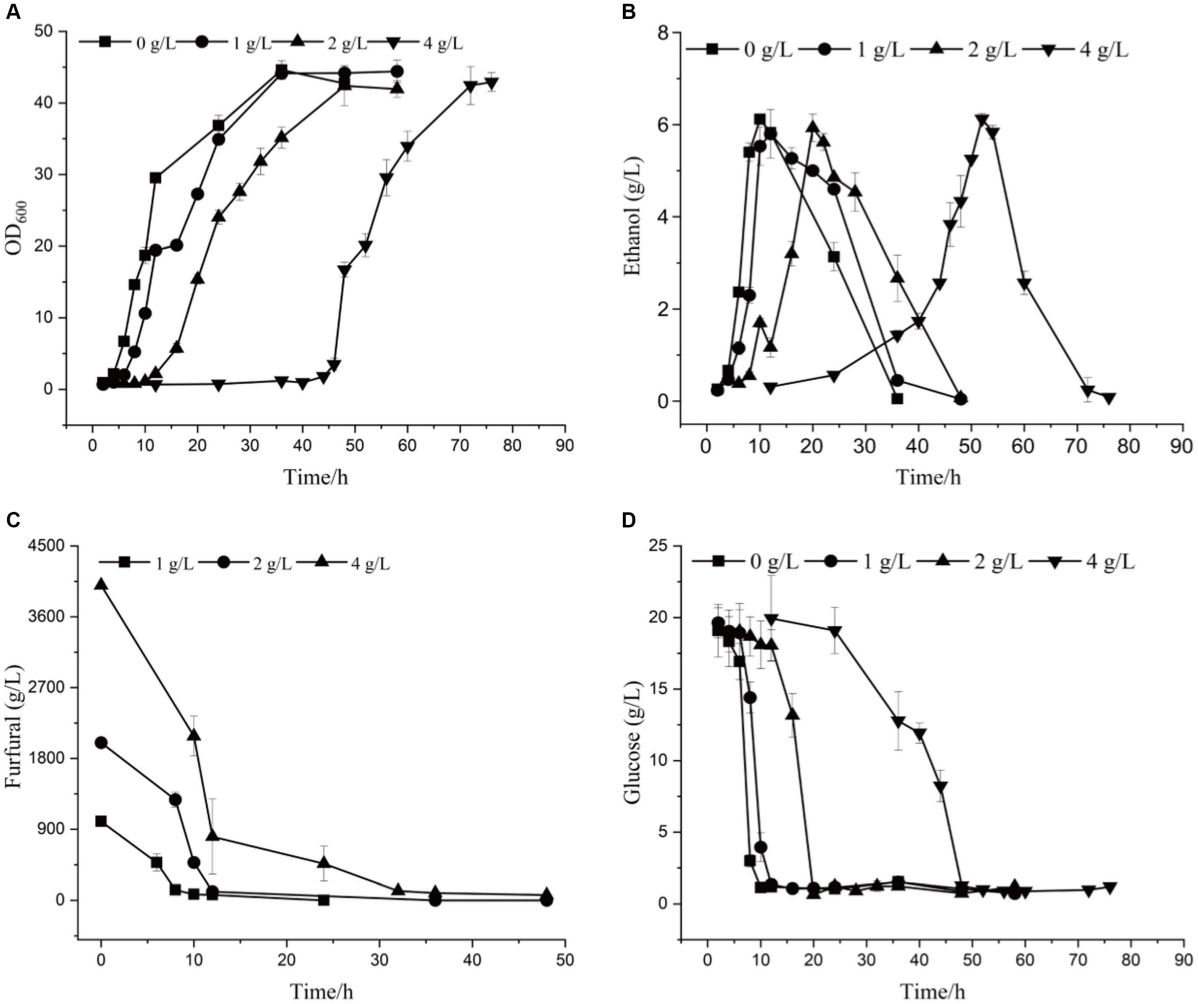
The growth curve **(A)**, ethanol yield **(B)**, furfural concentration **(C)**, and glucose content **(D)** of 12–1 strain under different furfural stress.

By comparing the differences in tolerance to furfural stress between the adapted strain and the wild-type strain, it was found that the starting strain did not grow on a YEPD solid plate containing 1.5 g/L furfural, while the evolutionary strain 12–1 could (Supplementary Figure S4). The results showed 12–1 strain showed the best performance under 4.0 g/L of furfural stress (Supplementary Figure S5). Compared with the original strain (Supplementary Figure S6; Supplementary Table S1), under 4.0 g/L of furfural treatment, the time to get to exponential phase was reduced by 36 h, and 7.02% more ethanol was obtained (Figure 2 and Table 3). The furfural was completely degraded after 48 h, indicating that the time required to degrade furfural was significantly shortened. Furthermore, the specific growth rate of 12–1 was 32.43% higher than that of *S. cerevisiae* CEN.PK113-7D, and the ethanol conversion rate was 6.67% higher. 1 g/L of furfural could reduce ethanol productivity of 12–1 strain obviously. 4 g/L of furfural could decrease ethanol productivity by 82%, indicating ethanol productivity was significantly decreased with the increase of furfural concentration. The reason was that during the lag phage, yeast cells needs to convert furfural into less toxic furfuryl alcohol, hardly produces ethanol, resulting in a decrease in ethanol productivity.

**Table 3:** Kinetic parameters of adaptive strains in YEPD medium.

Furfural (g/L)	Lag phase (h)	*μ* (/h)	*X*_max_ (g/L)	*Y*_X/S_ (mg/g)	EtOH_max_ (g/L)	*Y*_P/S_ (g/g)	Ethanol productivity (g/L/h)
0	2	0.105 ± 0.004	44.6 ± 1.31	2.6 ± 0.034	6.1 ± 0.02	0.34 ± 0.01	0.61 ± 0.02
1	4	0.079^**^ ± 0.005	44.4 ± 1.51	2.3^***^ ± 0.011	5.8 ± 0.53	0.30 ± 0.03	0.48 ± 0.05
2	10	0.079^**^ ± 0.002	42.4 ± 2.82	2.3^***^ ± 0.029	5.9 ± 0.30	0.30^**^ ± 0.01	0.25^***^ ± 0.03
4	44	0.049^***^ ± 0.001	42.9 ± 1.32	2.2^***^ ± 0.009	6.1 ± 0.12	0.32 ± 0.02	0.11^***^ ± 0.01

^*^(0.01 ≤ *p* < 0.05), ^**^(0.001 ≤ *p* < 0.01), ^***^(*p* < 0.001).

### 3.2 Whole genome sequencing of evolved mutants

**Figure 3:**
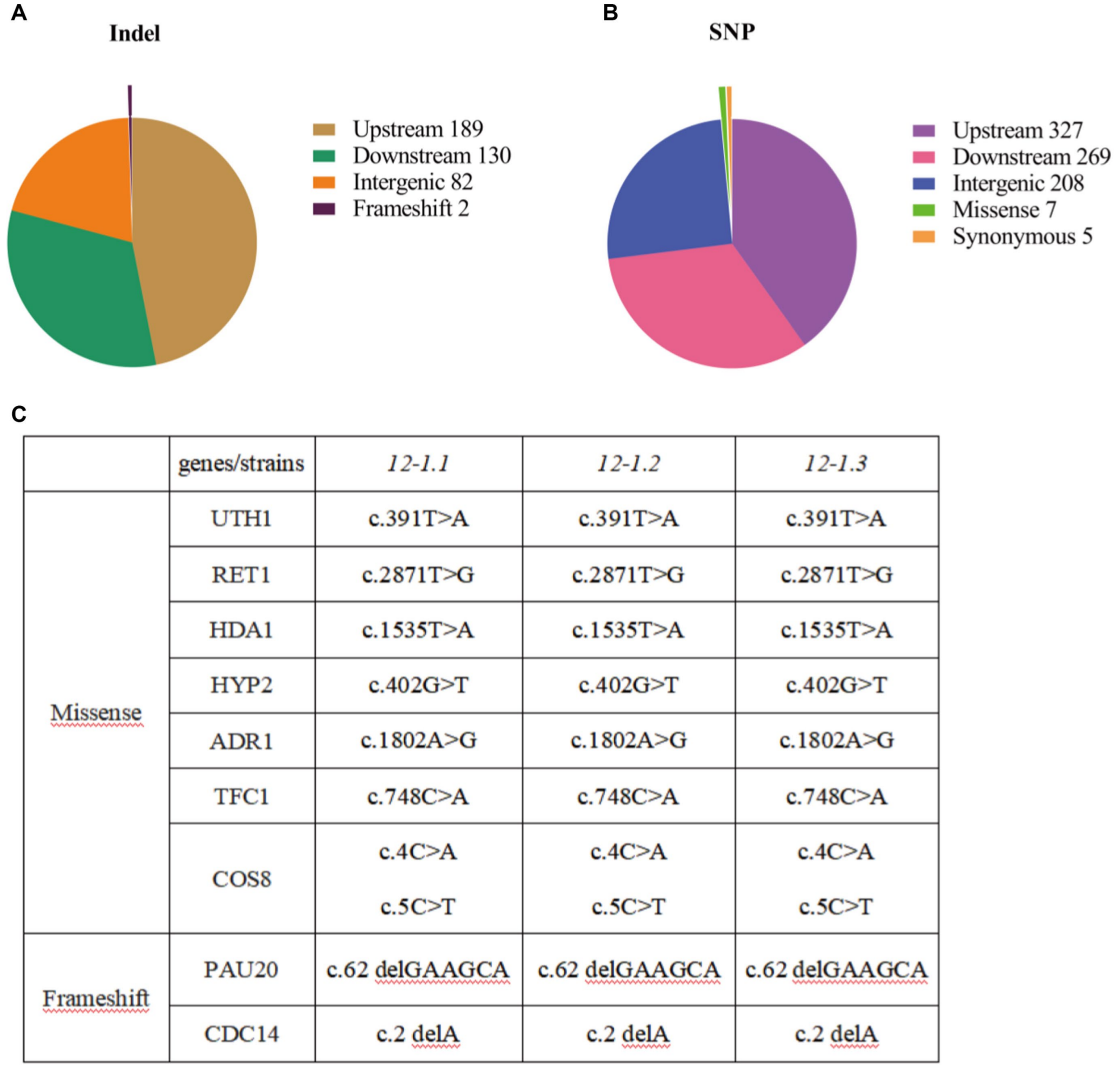
Results of whole genome sequencing. **(A)** Statistical results of single nucleotide insertion or deletion mutations in whole genome sequencing. **(B)** Statistical results of single nucleotide mutations in whole genome sequencing. **(C)** Statistical results of mutation types and mutation genes in whole genome sequencing.

In order to explore the changes in genes that enhance tolerance of 12–1 strain to furfural stress, the entire genome sequencing of 12–1 and CEN.PK113-7D strain were sequenced. A total of 1,219 mutations were obtained, including 403 single nucleotide insertion or deletion mutations (Indel) and 816 single nucleotide polymorphisms (SNP) (Figures 3A,B). It was suggested that the adaptation of *Saccharomyces cerevisiae* CEN.PK113-7D to furfural stress may be closely related to gene expression regulation. In addition, *Saccharomyces cerevisiae* can also adapt to furfural stress by changing the function of some proteins to promote their degradation (Zhao et al., 2015). The specific mutation sites of each mutanted gene were shown in Figure 3C. The genes with missense mutations were *UTH1*, *RET1*, *HDA1*, *HYP2*, *ADR1*, *TFC1*, and *COS8*, and the genes with frameshift mutations were *PAU20* and *CDC14,* respectively. In summary, strain 12–1 may enhance the adaptation to furfural stress by prolonging cell life, affecting autophagy, changing the structure and function of cell walls, and improving the viability of cells.

### 3.3 Effects of *ADR1* mutation on furfural tolerance of *Saccharomyces cerevisiae*

**Figure 4:**
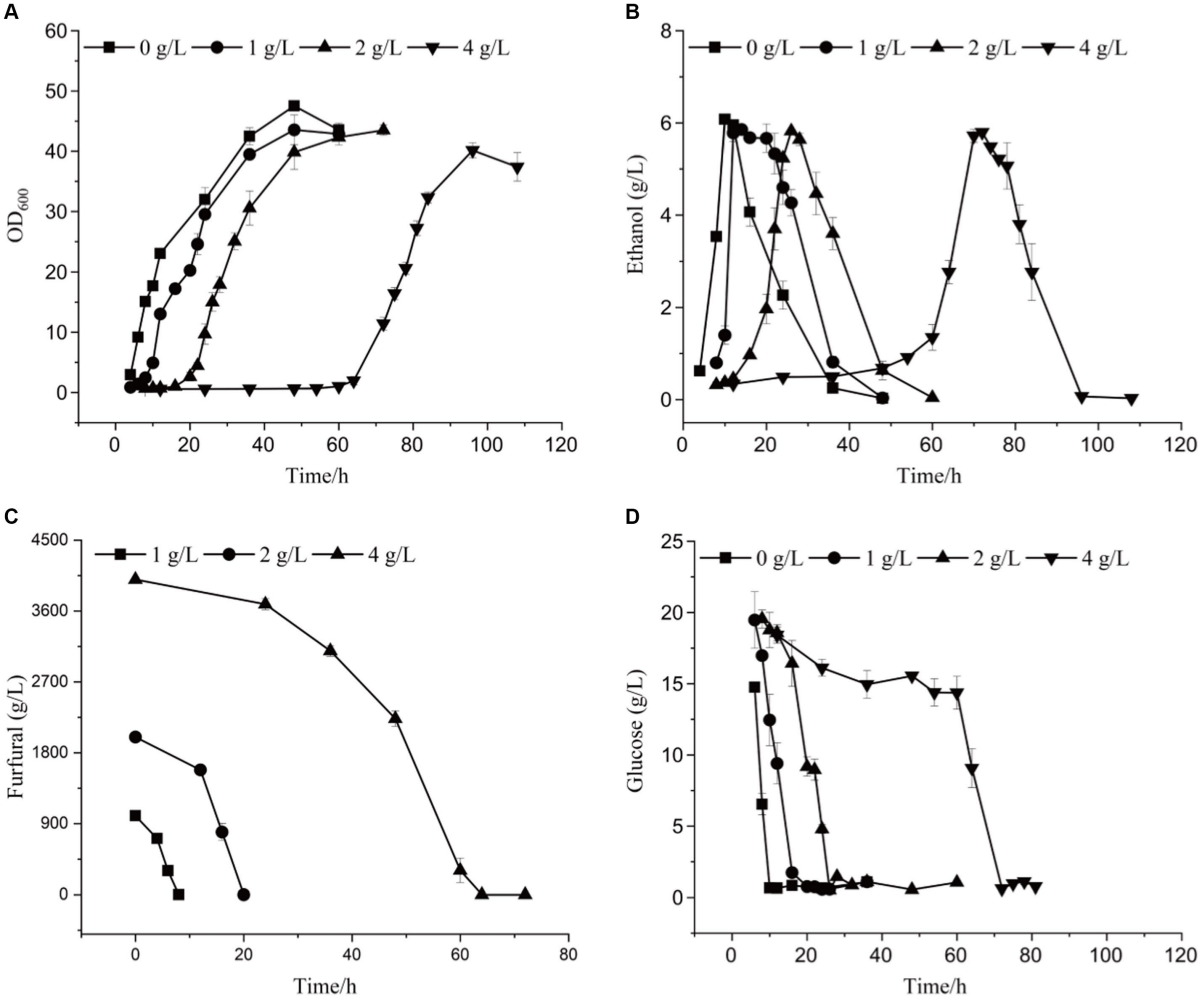
The growth curve **(A)**, ethanol yield **(B)**, furfural concentration **(C)**, and glucose content **(D)** of ADR1_1802 mutant under different furfural stress.

Based on the whole genome sequencing results, the *ADR1* gene mutation was constructed to obtain the engineered strain ADR1_ 1802, to explore the effect of ADR1 mutation on the growth and fermentation of *Saccharomyces cerevisiae* under furfural stress. Effects of different concentrations of furfural on the growth and ethanol production of ADR1_1802 mutant were determined, as shown in Figure 4, Supplementary Figure S7, and Table 4. Compared with the reference strain (*S. cerevisiae* CEN. PK113-5D), under 4 g/L furfural treatment, the lag phase was shortened by 20 h.

**Table 4:** Kinetic parameters of *ADR1_1802* mutant strain in YEPD medium.

Furfural (g/L)	Lag phase (h)	*μ* (/h)	*X*_max_ (g/L)	*Y*_X/S_ (mg/g)	EtOH_max_ (g/L)	*Y*_P/S_ (g/g)	Ethanol productivity (g/L/h)
0	2	0.080 ± 0.004	47.5 ± 0.39	3.3 ± 0.034	6.1 ± 0.03	0.43 ± 0.01	0.61 ± 0.03
1	6	0.078 ± 0.003	43.6 ± 2.47	2.3^***^ ± 0.096	5.9^**^ ± 0.04	0.31^*^ ± 0.05	0.49 ± 0.04
2	16	0.067^**^ ± 0.001	43.5^**^ ± 0.89	2.3^***^ ± 0.009	5.8^***^ ± 0.01	0.31^**^ ± 0.02	0.24^***^ ± 0.01
4	60	0.041^***^ ± 0.001	40.1^**^ ± 1.25	2.2^***^ ± 0.008	5.8^**^ ± 0.05	0.32^***^ ± 0.01	0.08^***^ ± 0.05

^*^(0.01 ≤ *p* < 0.05), ^**^(0.001 ≤ *p* < 0.01), ^***^(*p* < 0.001).

### 3.4 Effects of ADR1p mutation on physiological characteristics of *Saccharomyces cerevisiae* under furfural stress

**Figure 5:**
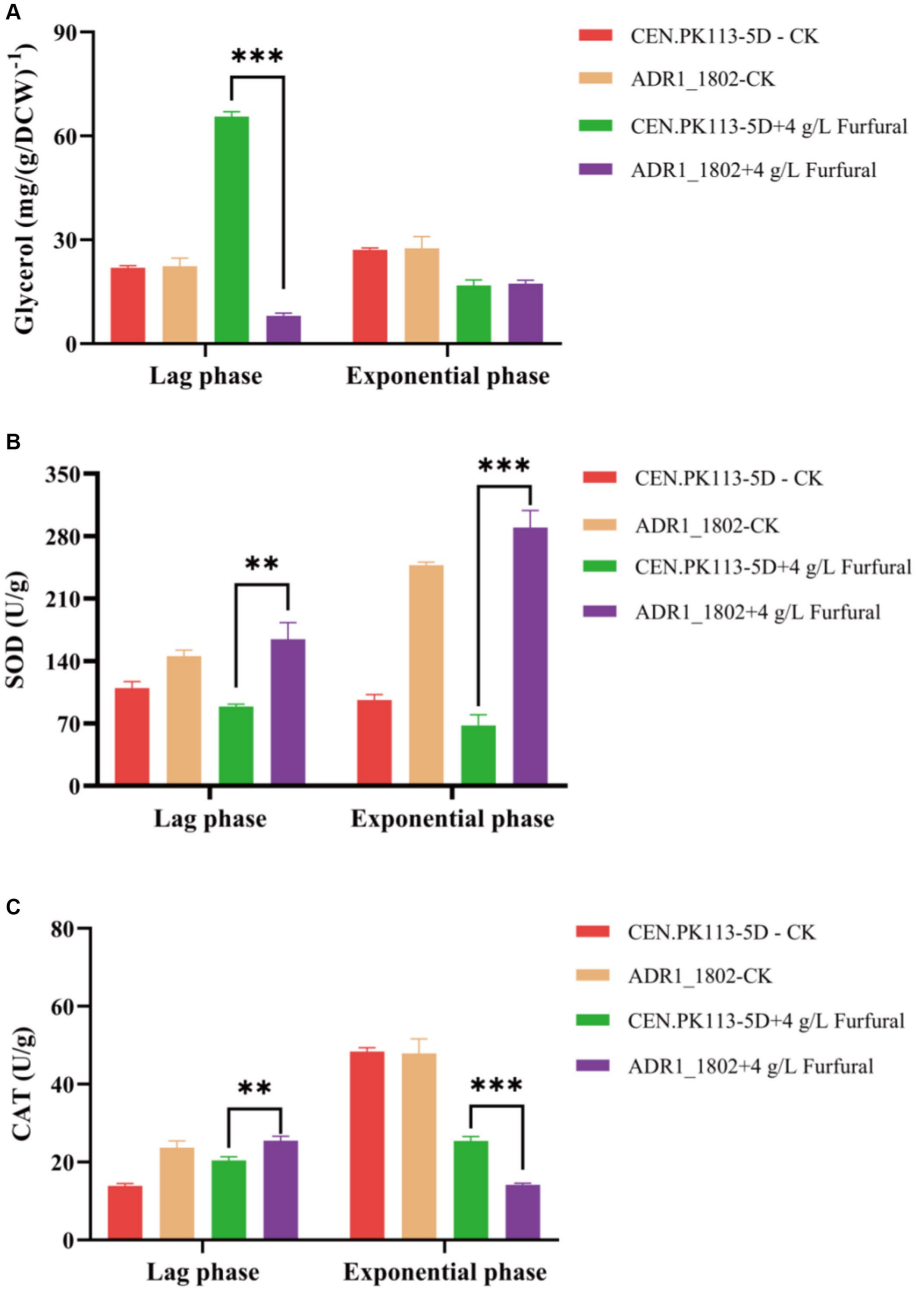
Comparison of mutant strain ADR1_1802 with reference strain of intracellular glycerol **(A)**, SOD **(B)** and CAT **(C)** enzyme activity under furfural stress. “**” (0.001 ≤ *p* < 0.01), “***” (*p* < 0.001).

Adr1p is essential for the transcription of genes required for glycerol utilization, and glycerol is the main osmotic protector for *Saccharomyces cerevisiae* (Hohmann, 2002). Glycerol plays an important role in yeast adaptation to changing conditions (Påhlman et al., 2000). As shown in Figure 5A, during the lag phase, the intracellular glycerol content of the ADR1_1802 mutant was 87.74% lower than that of the 5D strain, and there was no significant difference in the logarithmic phase. Based on this result, it was indicated that intracellular glycerol was applied to resist the toxicity of furfural in ADR1_ 1802 mutant.

Due to involvement of ADR1 in the expression of peroxisome genes, under high concentration of furfural (4 g/L) stress, the enzyme activities of CAT and SOD in ADR_1802 mutant and reference strain were determined and compared. As shown in Figures 5B,C, compared with reference strain, SOD enzyme activity was increased by 84.52% and CAT enzyme activity was increased by 24.72% during the lag phase in ADR_1802 mutant. Nakazawa et al. found that due to the high activity of CAT and SOD of strain IB1306, this strain effectively resisted the increase in ROS levels induced by H_2_O_2_ (Nakazawa et al., 2018). Overexpression of catalase increases tolerance to furfural and HMF (Kim and Hahn, 2013).

ADR1_1802 mutant strain utilizes the intracellular glycerol, high activity of CAT, and SOD to resist the toxicity of high concentrations of furfural.

### 3.5 Effect of ADR1p mutation on the expression of genes for furfural degradation

Furfural could be reduced to furfuryl alcohol in *Saccharomyces cerevisiae* with NAD (P) H (Liu et al., 2008). From above results, it can be seen that the time required for the degradation of furfural by ADR1_1802 strain was significantly shorter than that of the reference strain, and the lag phase was shortened obviously. Therefore, the transcription level of genes in ADR1_1802 strain and reference strain related to the furfural degradation was analyzed and compared (Supplementary Figure S8).

**Figure 6:**
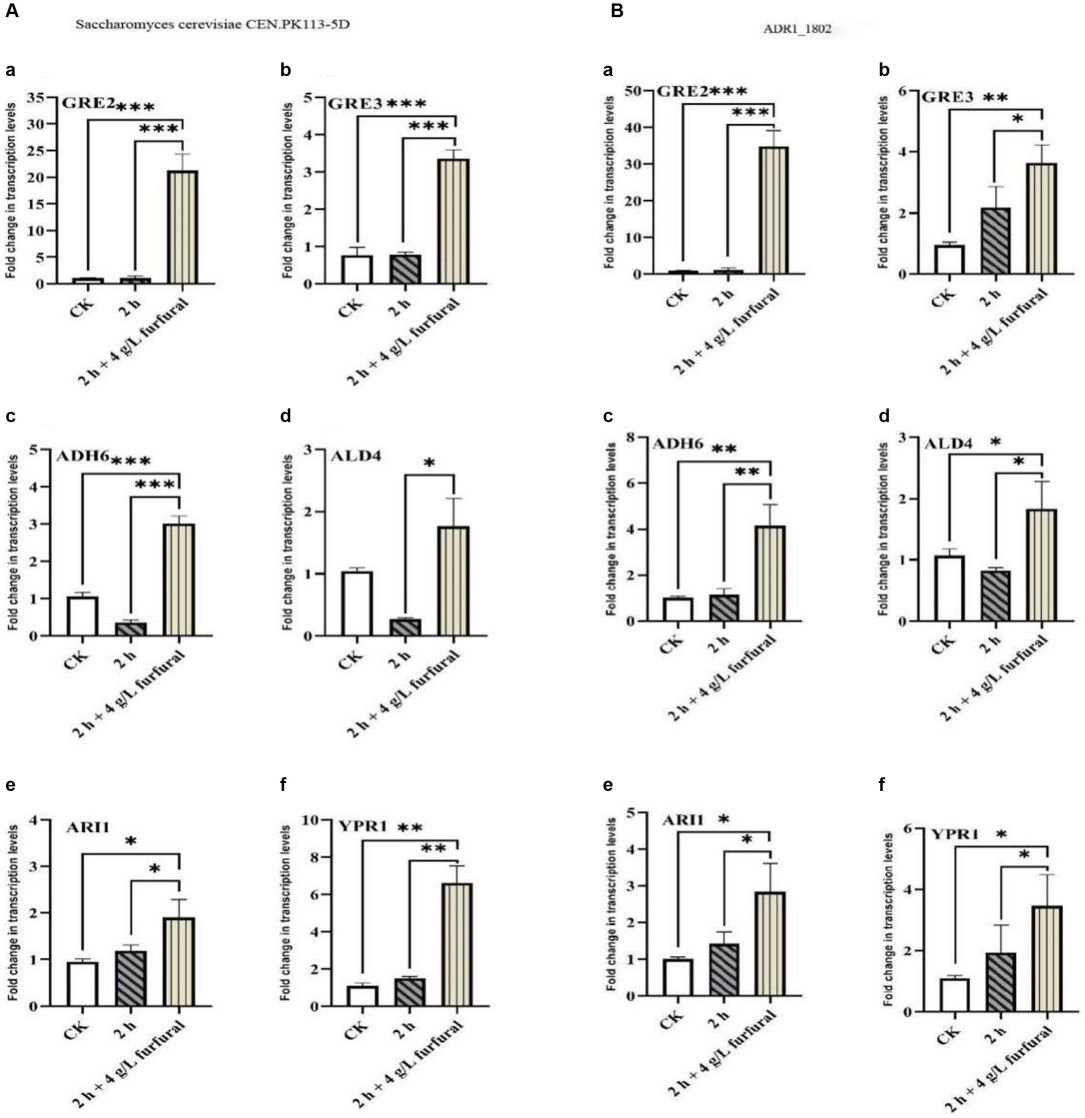
Transcription levels of *ADH6*, *ALD4*, *GRE2*, *GRE3*, *YPR1*, and *ARI1* in response to furfural. “*” (0.01 ≤ *p* < 0.05), “**” (0.001 ≤ *p* < 0.01), and “***” (*p* < 0.001).

As shown in Figure 6, the results of RT-PCR showed that, the transcription levels of *ADH6*, *ALD4*, *GRE2*, *GRE3*, *YPR1*, and *ARI1* genes in *S. cerevisiae* CEN. PK113-5D strain increased by 2.87, 1.69, 20.04, 4.37, 6.06, and 2.01 times, respectively, compared with control. While in ADR1_1802 mutant strain, they were upregulated by 4.16, 1.71, 35.80, 3.83, 3.15, and 2.84 times, respectively. It could conclude that *GRE2* was a key gene for the reduction of furfural. Under 4 g/L furfural treatment, compared with reference strain, transcription level of *GRE2* in ADR1_1802 mutant was increased by 53.69%, and that of *ADH6* was increased by 44.95%.

## 4 Discussion

After ALE by accumulating spontaneous mutations generation after generation under specific pressure of furfural, a modified microbial strain is obtained through forced selection of specific phenotypes. After a long period of adaptive laboratory evolution, this strain continuously adapts and evolves in a culture medium with gradually increasing furfural concentration, resulting in stains with faster growth and enhanced tolerance to furfural stress.

It was suggested that the adaptation of *Saccharomyces cerevisiae* CEN.PK113-7D to furfural stress may be closely related to gene expression regulation. For *Saccharomyces cerevisiae*, Pau protein may play a role in adapting to stress, which could be induced to express under low temperature and anaerobic conditions (Luo and Van Vuuren, 2009). The regulation of sphingolipids synthesis by *COS8* could help cells obtain the ability to integrate signals from the pheromone, osmolarity, and TOR pathways to modify membrane structure (Bailly-Bechet et al., 2011). Genes *TFC1*, *HYP2*, and RET1 are mainly involved in cell proliferation and growth, and are crucial for cell survival (Swanson et al., 1991; Bobkova et al., 1999). The proteins Uth1p, Hda1p, Cdc14p, and Adr1p are closely related with oxidative stress responses, cell lifespan, and autophagy in yeast (Valenzano et al., 2006). Mutations in these genes may alter intracellular oxidative stress levels, extend cell lifespan, and change the structure and function of cell walls, thereby improving the adaptability of *Saccharomyces cerevisiae* to furfural stress.

Adr1p is transcription factor that is required for the transcription of glucose inhibitory gene *ADH2*, peroxisome protein gene, and genes required for ethanol, glycerol, and fatty acid utilization. When glucose is absent, *Saccharomyces cerevisiae* can also effectively grow in carbon sources such as glycerol, acetate, ethanol, or oleate. This requires metabolic rearrangement, and involves several transcription factors, of which Adr1 is the most critical one (Moreno-Cermeño et al., 2013). Young et al. found that peroxisome genes were significantly upregulated under derepressing conditions due to the loss of Med14 activity, which were dependent on the Adr1 and Oaf1/Pip2 transcription complexes (Young et al., 2009). The sequencing analysis results in this study showed that there was a missense mutation in the *ADR1* gene of 12–1 strain (lysine at 601 changed to arginine).

After ADR1p mutation on *Saccharomyces cerevisiae*, it was found that during the lag phase, the intracellular glycerol content of the ADR1_1802 mutant was 87.74% lower than that of reference strain. Previous studies by Ask et al. found that the addition of 3.9 g/L HMF and 1.2 g/L furfural to *Saccharomyces cerevisiae* resulted in significant physiological reactions. The specific growth rate and glycerol production was decreased significantly (Ask et al., 2013). The physiological characteristics of a new heat-resistant strain (LBGA-01) was found by Cleiton et al., which can grow at 40°C and is more resistant to sucrose, furfural, and ethanol than industrial strains. At 40°C, gene expression related to the formation of glycerol (*GPD2*) is reduced (Prado et al., 2020). Furthermore, Reactive oxygen species (ROS) generated during metabolic process, includes hydrogen peroxide (H_2_O_2_), hydroxyl radicals, and superoxide anions, can damage proteins, lipids, carbohydrates, and DNA (Bora et al., 2018). Under high concentration of furfural (4 g/L) stress, ADR1_1802 mutant strain showed higher CAT and SOD enzyme activity than the wild-type strain in lag phage, which is consistent with previous studies (Nakazawa et al., 2018). Although CAT activity in the mutant strain in exponential phase was lower than that of the wild-type, lag phage is more important, during which furfural was degraded by cells metabolism to less toxic compounds.

Earlier studies indicated that overexpression of *ADH6* in *Saccharomyces cerevisiae* enhances the *in situ* detoxification furfural produced during biomass pretreatment (Quarterman et al., 2018). Furthermore, the HMF conversion rate *in vivo* of *S. cerevisiae* CEN.PK 113-5D overexpressing *ADH6* was significantly increased, indicating that overexpression indeed increased reduction capacity (Petersson et al., 2006). The *GRE2* gene of *Scheffersomyces (Pichia) stipitis* is highly induced under furfural and HMF stress, which can enhance tolerant ability of *S. cerevisiae* to furfural after overexpression (Wang et al., 2016).

In summary, the expression of genes related to furfural tolerance such as *GRE2* and *ADH6* were increased for the reduction of furfural to furfural alcohol in ADR1 mutation strain, providing sufficient NAD (P)(+) for NAD (P) H regeneration, thereby enhancing resistance of *ADR1_1802* mutant to furfural stress.

## 5 Conclusion

The present study involved screening 12–1 strains of *Saccharomyces cerevisiae* by laboratory adaptive evolution to improve ethanol production and shorten the lag phase. Whole genome sequencing results demonstrated that*ADR1* genecan be mutated to get ADR1_1802 mutant by CRISPR/Cas9 technology, which exhibited increased resistance to furfural stress comparable fermentation performance with reference strain. These findings suggested the potential for genetic engineering to increase the furfural tolerance of *Saccharomyces cerevisiae*.

## Data availability statement

The original contributions presented in the study are included in the article/Supplementary material, further inquiries can be directed to the corresponding author. Sequence data associated with this project have been deposited in the NCBI (PRJNA1049636).

## Author contributions

LY: Writing – original draft. YJ: Formal analysis, Methodology, Writing – original draft. QZ: Investigation, Writing – review & editing. XZ: Writing – review & editing. HY: Writing – review & editing. JD: Writing – review & editing. XC: Funding acquisition, Supervision, Writing – review & editing.
