# Supplementary material for: Adaptive laboratory evolution to obtain furfural tolerant Saccharomyces cerevisiae for bioethanol production and the underlying mechanism
Source: Front Microbiol. 2024 Jan 4;14:1333777. doi: 10.3389/fmicb.2023.1333777 (PMC10794740; doi:10.3389/fmicb.2023.1333777)
Supplement: Supplementary file 1 [file Data_Sheet_1.docx]

Adaptive Laboratory Evolution to Obtain Furfural Tolerant *Saccharomyces cerevisiae* for bioethanol production and the underlying Mechanism

Lan Yao,^1,a^ Youpiao Jia,^1,a^ Qingyan Zhang, ^a^ Xueyun Zheng, ^a^ Haitao Yang, ^b^ Jun Dai, ^a^ Xiong Chen *^,a^

^a^Key Laboratory of Fermentation Engineering (Ministry of Education), Cooperative Innovation Center of Industrial Fermentation (Ministry of Education & Hubei Province), College of Bioengineering, Hubei University of Technology, 28th of Nanli Road, Wuhan 430068, China

^b^Hubei Provincial Key Laboratory of Green Materials for Light Industry, Hubei

University of Technology, 28th of Nanli Road, Wuhan 430068, China

*Correspondence: cx163_qx@163.com.

^1^The authors contributed equally to this paper


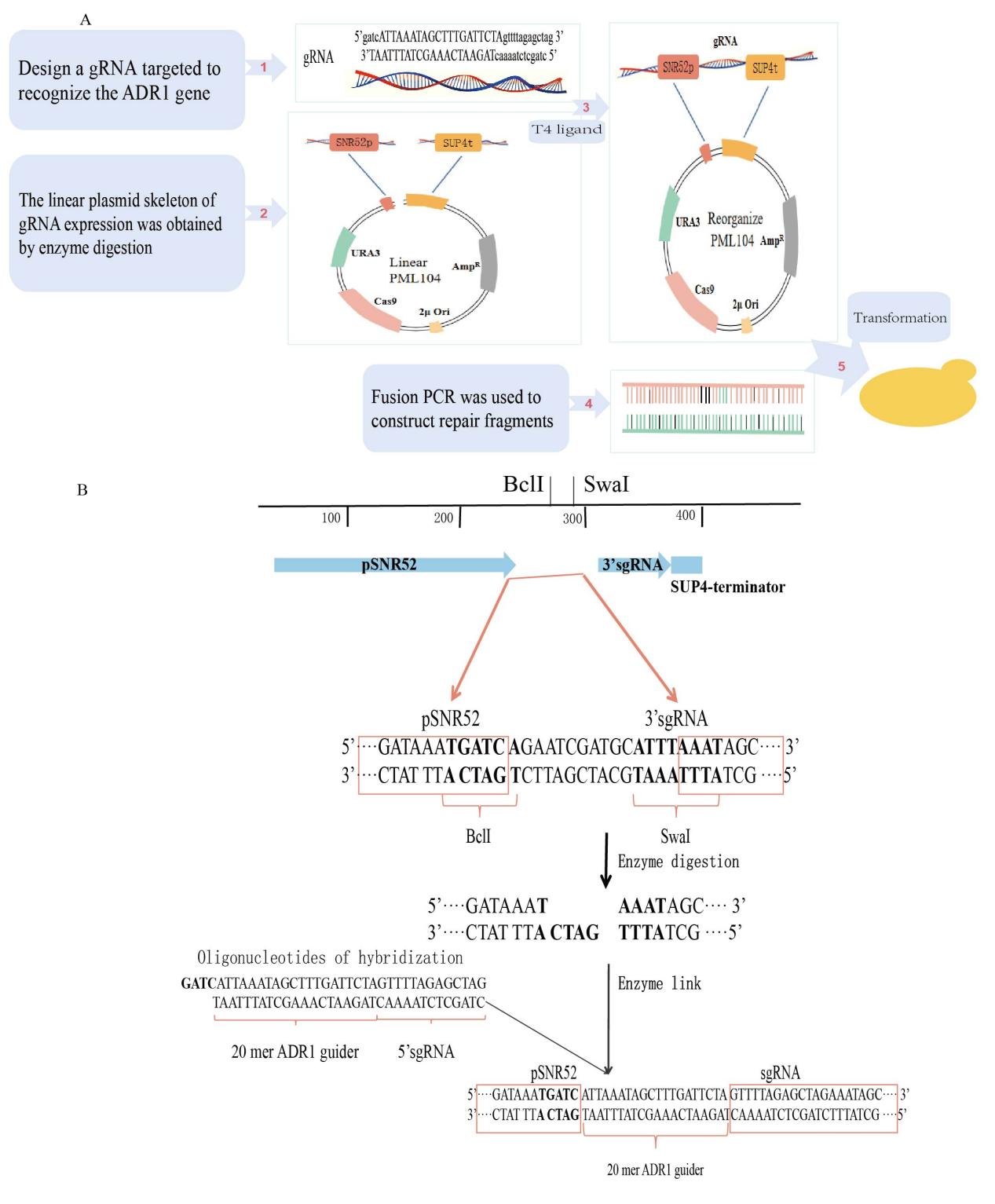


Fig.S1. CRISPR/Cas9 gene mutation process based on pML104 plasmid


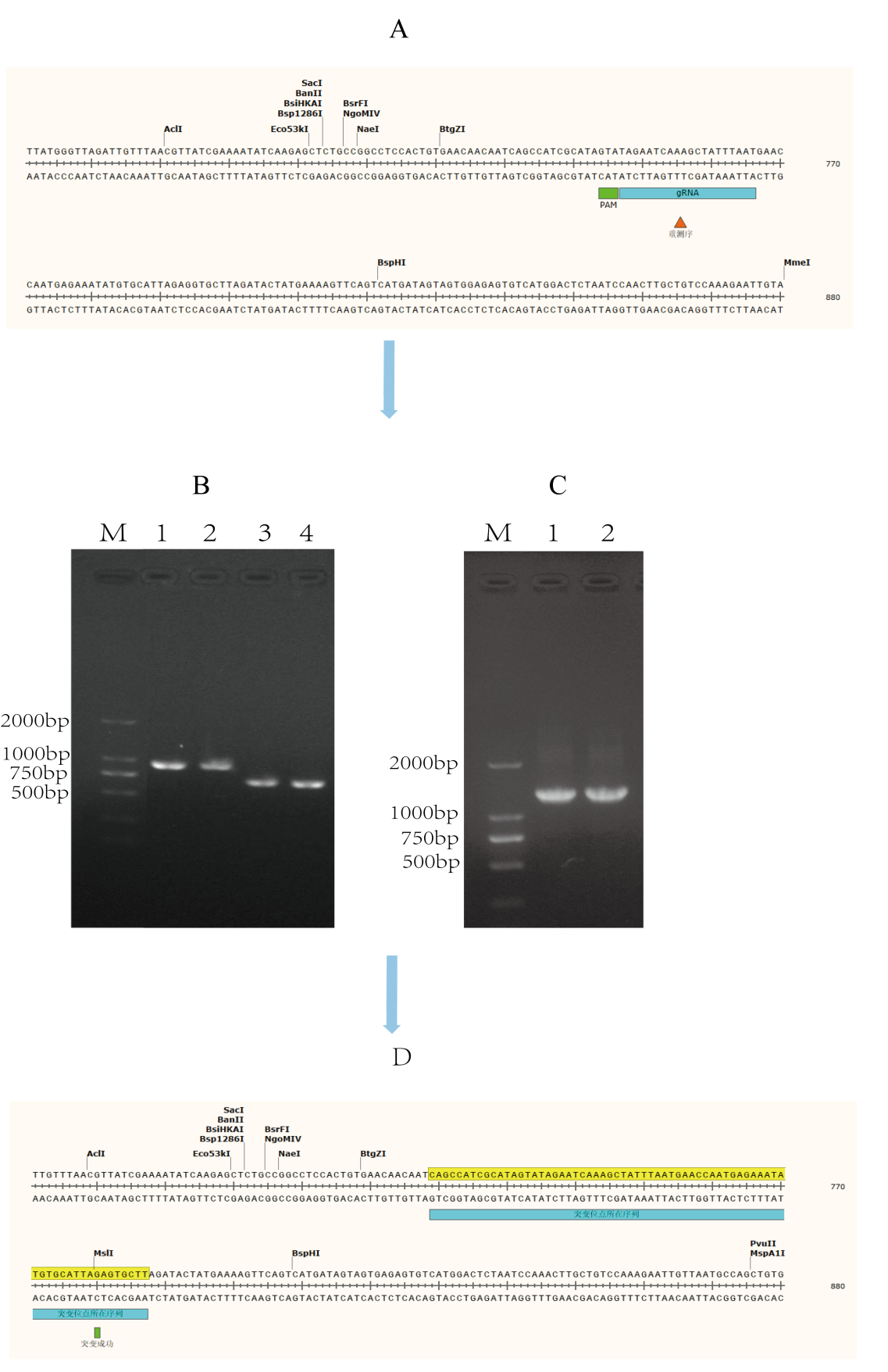


Fig. S2. The results of engineered strain by CRISPR/Cas9, A is the sequencing result of the successfully constructed recombinant plasmid pML104-adr1gRNA, B is the PCR amplification homologous arm, C is the Donor fragment constructed by fusion PCR, and D is the sequencing result of successfully mutated ADR1_1802


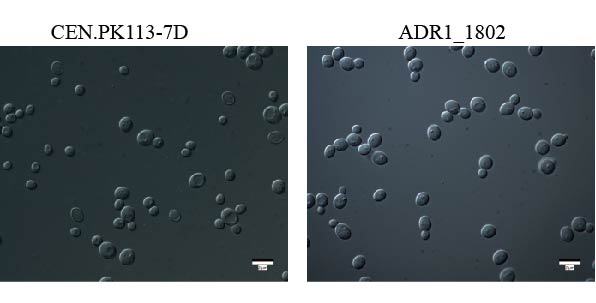


Fig. S3 Images of strains in the presence of 4 g/L of furfural before and after ALE


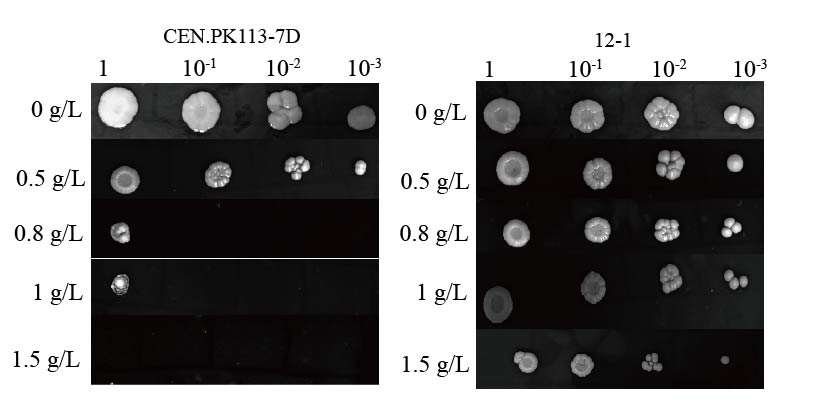


Fig. S4 Spot assay of strains before and after ALE in the presence of different furfural concentrations


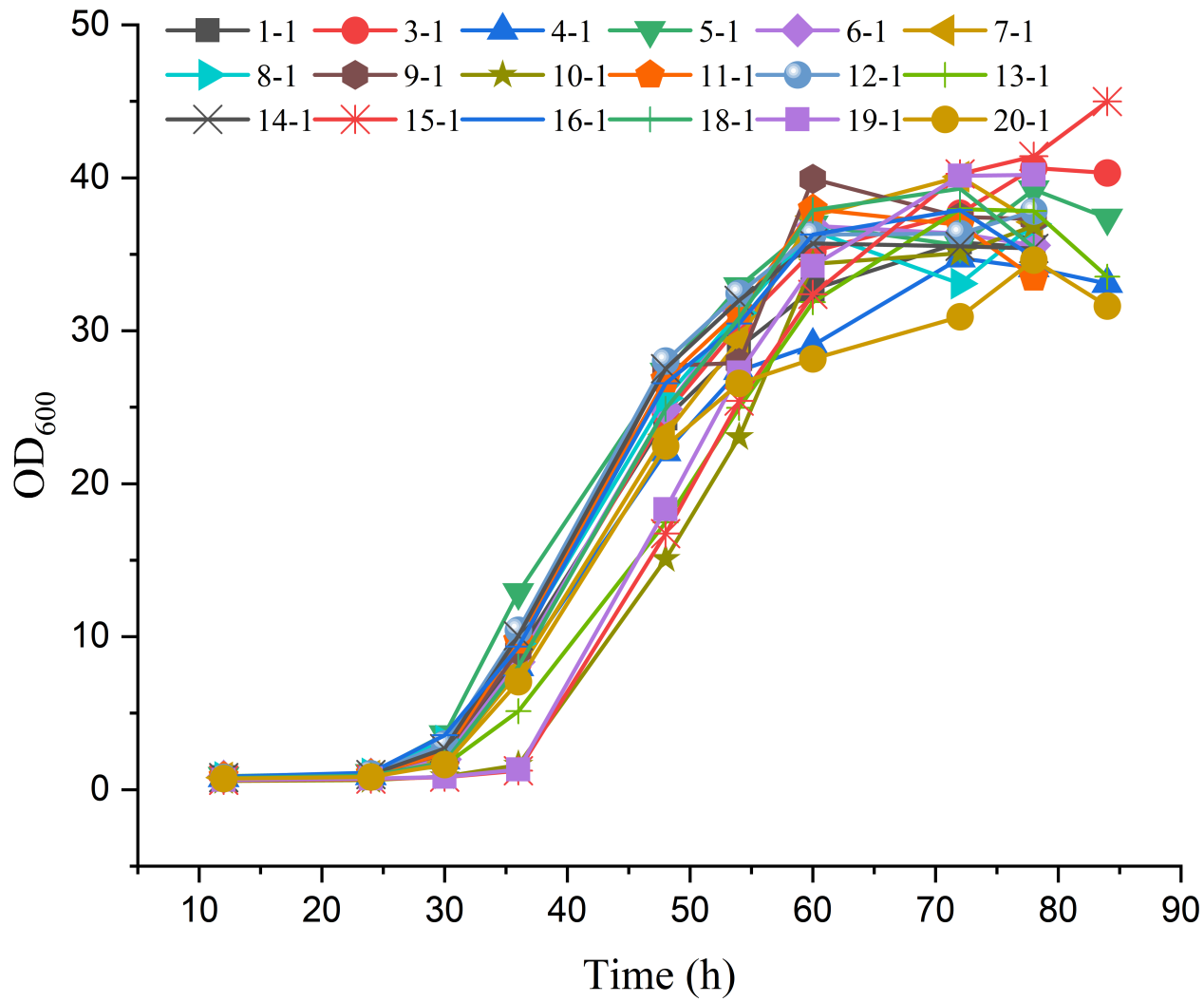


Fig. S5. The growth curve of adaptive strains under furfural stress (4g/L)


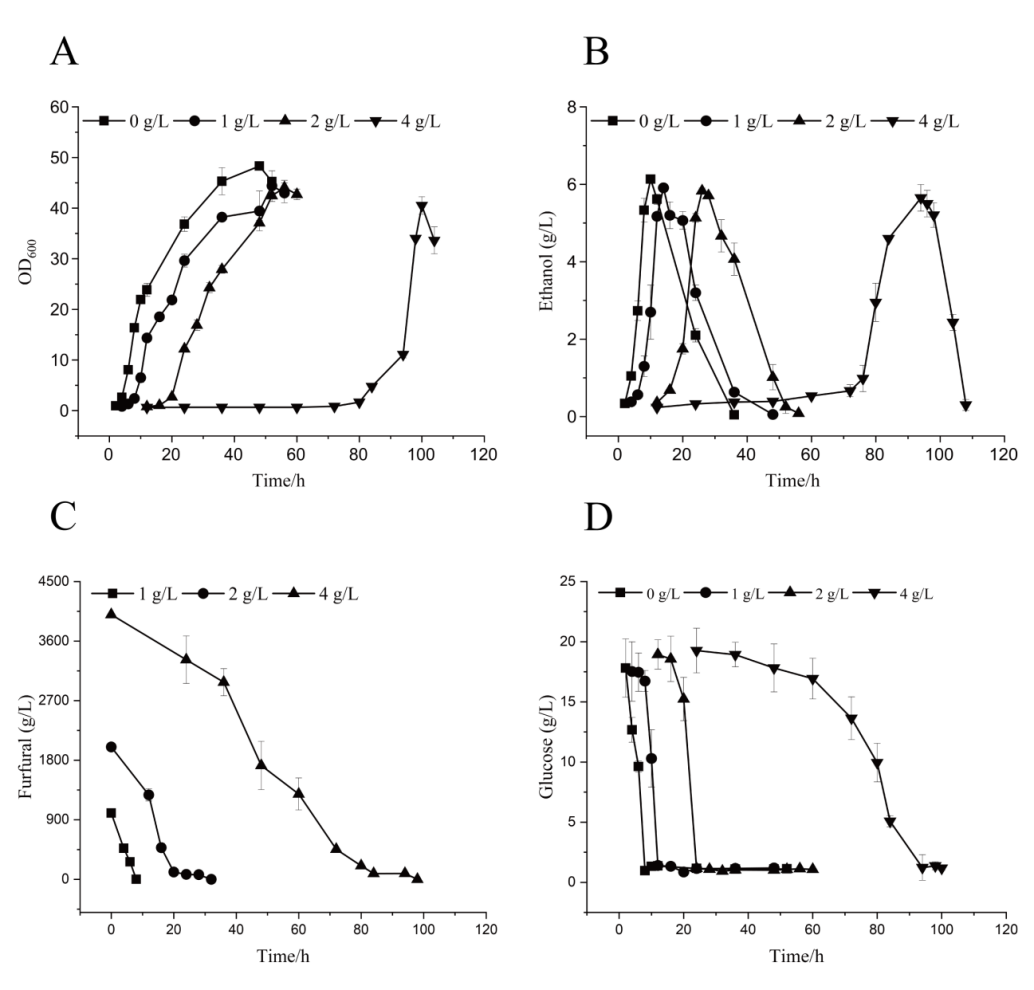


Fig. S6 The growth curve (A), ethanol yield (B), furfural concentration (C), and glucose content (D) of origin strain under different furfural stress


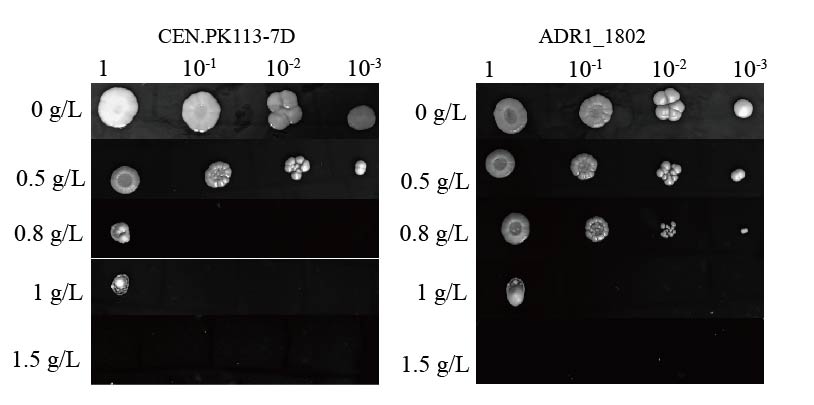


Fig. S7 Spot assay of strains before and after *ADR1* Mutation in the presence of different furfural concentrations


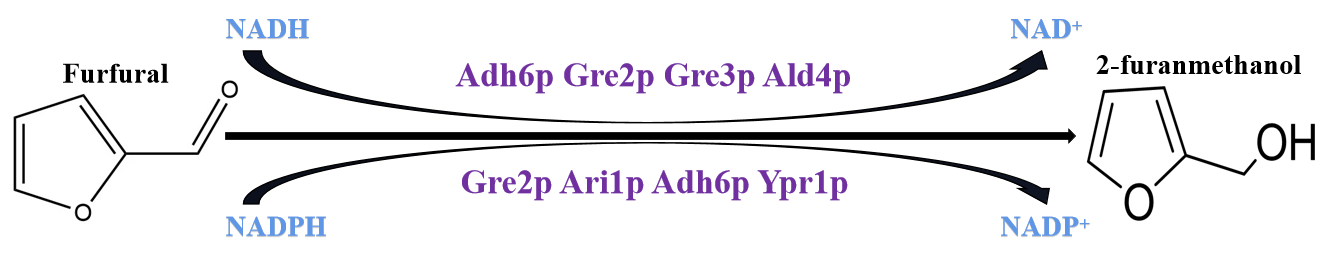


Fig. S8 Genes related to furfural degradation in *Saccharomyces cerevisiae*

Table S1. Kinetic parameters of origin strains in YEPD medium

| Furfural  (g/L) | Lag phase  (h) | *μ* (/h) | *X*_max_ (g/L) | *Y*_X/S_ (mg/g) | EtOH_max_ (g/L) | *Y*_P/S_ (g/g) | Ethano productivity  (g/L/h) |
| --- | --- | --- | --- | --- | --- | --- | --- |
| 0 | 2 | 0.081±0.005 | 48.3±0.45 | 2.8±0.025 | 6.1±0.02 | 0.36±0.02 | 0.61±0.02 |
| 1 | 6 | 0.073±0.003 | 44.4*±1.55 | 2.6**±0.027 | 5.9*±0.08 | 0.35±0.02 | 0.49±0.08 |
| 2 | 16 | 0.067**±0.001 | 44.1**±1.44 | 2.4***±0.023 | 5.8**±0.07 | 0.32*±0.01 | 0.24^***^±0.07 |
| 4 | 80 | 0.037***±0.001 | 40.5**±1.73 | 2.4***±0.026 | 5.7±0.34 | 0.30*±0.01 | 0.06^***^±0.03 |

"*" (0.01 ≤ P<0.05), "**" (0.001 ≤ P<0.01), and "***" (P<0.001).
